# Functionalized graphene-based biomimetic microsensor interfacing with living cells to sensitively monitor nitric oxide release†
†Electronic supplementary information (ESI) available: Detailed experimental procedures and materials; Fig. S1–S10 and Table S1. See DOI: 10.1039/c4sc03123g



**DOI:** 10.1039/c4sc03123g

**Published:** 2015-01-28

**Authors:** Yan-Ling Liu, Xue-Ying Wang, Jia-Quan Xu, Chong Xiao, Yan-Hong Liu, Xin-Wei Zhang, Jun-Tao Liu, Wei-Hua Huang

**Affiliations:** a Key Laboratory of Analytical Chemistry for Biology and Medicine (Ministry of Education) , College of Chemistry and Molecular Sciences , Wuhan University , Wuhan , 430072 , China . Email: whhuang@whu.edu.cn ; Fax: +86-27-68754067 ; Tel: +86-27-68752149

## Abstract

It is a great challenge to develop electrochemical sensors with superior sensitivity that concurrently possess high biocompatibility for monitoring at the single cell level. Herein we report a novel and reusable biomimetic micro-electrochemical sensor array with nitric oxide (NO) sensing-interface based on metalloporphyrin and 3-aminophenylboronic acid (APBA) co-functionalized reduced graphene oxide (rGO). The assembling of high specificity catalytic but semi-conductive metalloporphyrin with high electric conductive rGO confers the sensor with sub-nanomolar sensitivity. Further coupling with the small cell-adhesive molecule APBA obviously enhances the cytocompatibility of the microsensor without diminishing the sensitivity, while the reversible reactivity between APBA and cell membrane carbohydrates allows practical reusability. The microsensor was successfully used to sensitively monitor, in real-time, the release of NO molecules from human endothelial cells being cultured directly on the sensor. This demonstrates its potential application in the detection of NO with very low bioactive concentrations for the better understanding of its physiological function and for medical tracking of patient states.

## Abstract 2

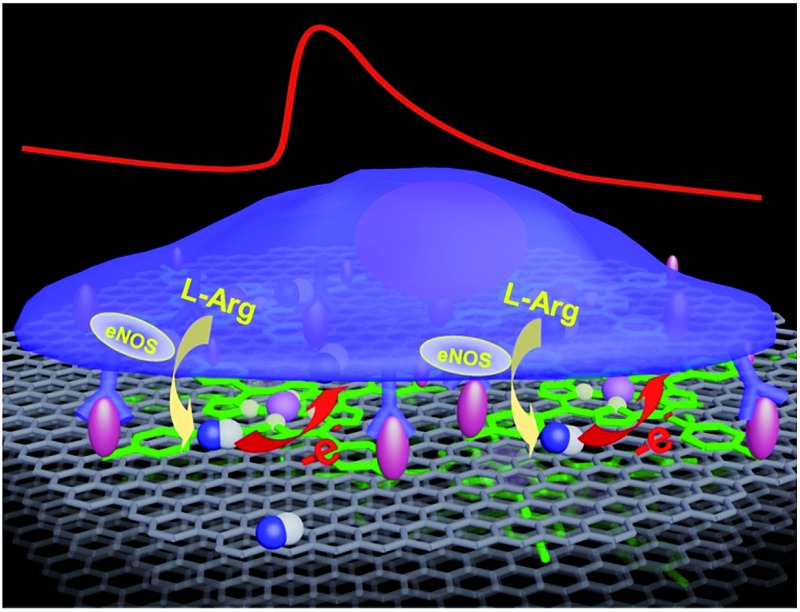
We present a biomimetic and reusable microsensor with sub-nanomolar sensitivity by elaboratly functionalizing graphene for monitoring NO release in real-time.

## Introduction

Nitric oxide (NO) has attracted intense interest due to its diverse and vital role in the regulation of physiological processes, such as cardiovascular systems, neurotransmission, immune responses and angiogenesis.^1–6^ The quantification of NO *in vivo* is essential to comprehensively unravel its function in physiology. However, measuring NO in biological systems is challenging due to its short half-life (<10 s), trace level (physiological range of NO bioactivity is as low as 100 pM or below) and the presence of complex interfering species.^7–11^ Methods commonly available, including chemiluminescence, absorbance, fluorescence and electron paramagnetic resonance spectroscopy,^12–15^ suffer from high costs or complicated pretreatments that restrict real-time measurement. Although newly developed techniques such as field-effect transistors or surface-enhanced Raman spectroscopy allow for the real-time detection of NO outside or inside living cells,^16,17^ an electrochemical NO sensor based on microelectrodes can be a powerful tool for the direct and real-time monitoring of NO release in physiology due to the ultra-fast response time, high-spatial resolution and non-invasive size.^7,18–22^

In recent times, great efforts have been made to ensure that electrochemical sensors have sufficient sensitivity and selectivity to accurately detect NO. To improve sensor sensitivity, different types of catalytic materials, such as metalloporphyrins (phthalocyanine and salen),^23–25^ carbon nanotubes,^26^ metal nanoparticles and nanowires,^27–29^ have been employed to develop sensitive NO sensors. With its unique properties, graphene has emerged as a rising star material and it has advanced the development of electrochemistry.^30–32^ Recently, graphene has shown a great potential for the sensitive detection of NO.^33–35^ Moreover, enhanced selectivity was achieved by coating a permselective membrane onto the sensor.^7,21,22^ Nafion, Teflon and *o*-phenylenediamine are often used to restrict the diffusion of interferent molecules (*e.g.*, nitrite, ascorbic acid and dopamine) to the electrode surface.^23,36,37^

In comparison with isolated cells and the above electrode configuration, culturing cells on microelectrodes could reconstruct the *in vivo* interface between NO emitting cells and their receptor cells (*e.g.*, endothelium and smooth muscle cells) better, and the monitoring of NO from the tightly attached cells could therefore model real physiological conditions more closely. In this case, biocompatibility is a critical issue that should be seriously considered. Pre-coating with positively charged polyelectrolytes (*e.g.*, poly-l-lysine^38^) or an extracellular matrix (*e.g.*, laminin^39^ and collagen^40^) is typically utilized to boost cell attachment. However, the additional polymer and extracellular matrix for selectivity or biocompatibility enhancement will actually decrease electrode response.^7,17,41^ It was also quite difficult to coat very small or irregularly shaped electrode surfaces in a controlled way. Recently, small cell-adhesive molecules, such as RGD and monosaccharides,^34,41^ have been explored for interfacing electrodes with living cells, providing an approach to fabricate biocompatible sensors.

**Scheme 1:**
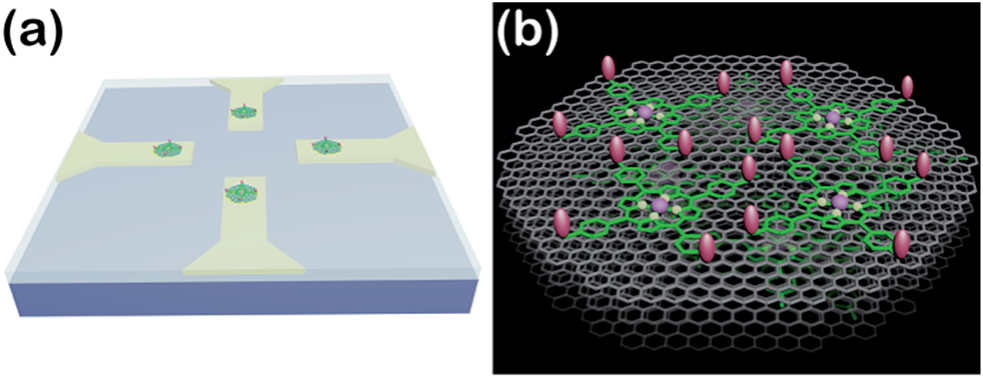
(a) Schematic of the APBA/FGHNs/ITO microelectrode array (only 4 microelectrodes are shown here for simplicity). (b) Schematic illustration of APBA/FGHNs in the active electrode area, and the gray, green and pink features represent rGO, FeTCP and APBA, respectively.

So far, great success has been achieved in developing NO electrochemical sensors with sensitivities above nanomolar concentrations. However, the construction of sensing-interfaces with sub-nanomolar to picomolar sensitivities that concurrently possess excellent cytocompatibility and high selectivity remains a great challenge, especially for the high throughput detection at single cell levels (*i.e.*, single or a few cells). Herein, we have developed a multifunctional microsensor array for the detection of NO from several cells. Inspired by the excellent catalysis of NO electrooxidation by metalloporphyrins and the two-dimensional nanostructure and high electric conductivity of graphene, we prepared novel hybrid nanosheets, based on Fe(iii) *meso*-tetra (4-carboxyphenyl) porphyrin (FeTCP) and reduced graphene oxide (rGO), through π–π interactions, which were then deposited onto an ITO microelectrode array *via* electrophoretic deposition. These nanosheets, named FGHNs, were further functionalized covalently with a small cell-adhesive molecule, 3-aminophenylboronic acid (APBA). This can react with the 1,2- or 1,3-diols in carbohydrate moieties that exist largely on cell membranes^42–45^ to provide the sensor with good cytocompatibility. The as-prepared microsensor array and the electroactive area are shown schematically in Scheme 1. The microsensor demonstrated exceptional sensitivity and selectivity to NO with a detection limit of 55 pM in PBS and 90 pM in a cell medium. Human umbilical vein endothelial cells (HUVECs) can adhere and proliferate well on the electrode surface, therefore promoting interfaces with the living cells being cultured thereon and high sensitive real-time monitoring of NO release.

## Results and discussion

### Preparation and characterization of the FGHNs sheets

**Fig. 1:**
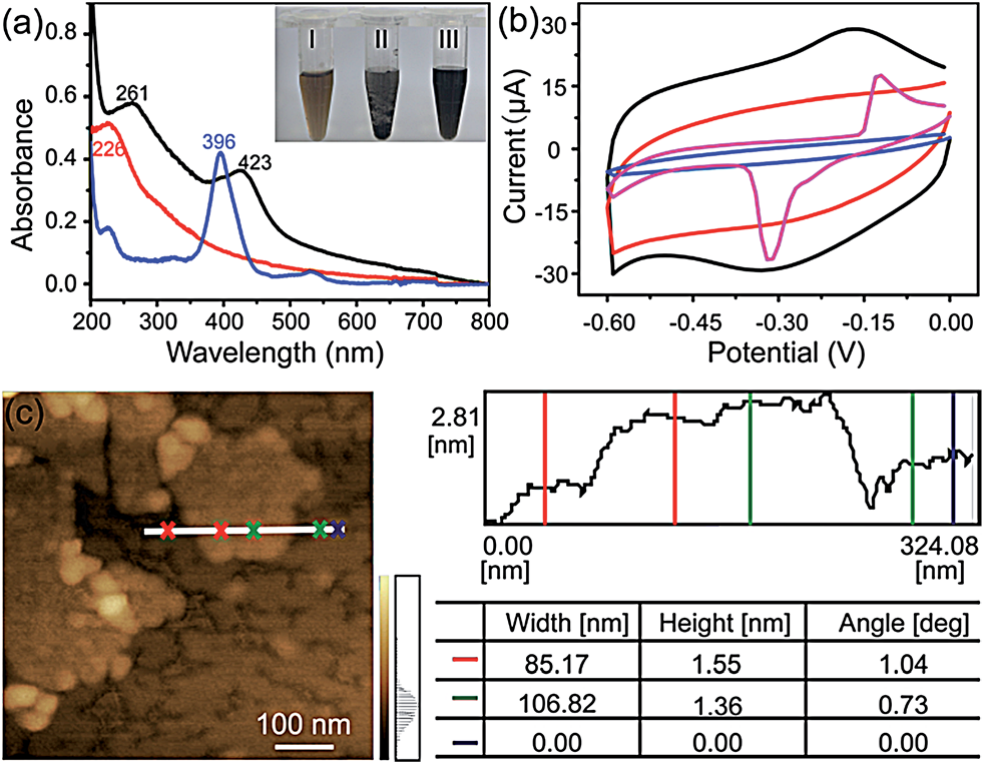
(a) UV-visible spectra of FeTCP solution (blue line), GO suspension (red line) and FGHNs suspension (black line). Inset: photographs of GO (I), rGO (II) and FGHNs (III) dispersed in water. (b) Cyclic voltammograms of bare ITO (blue line), FeTCP/ITO (purple line), rGO/ITO (red line), FGHNs/ITO (black line) in 0.1 M PBS saturated with N_2_ at a scan rate of 50 mV s^–1^. (c) AFM image of FGHNs.

The FGHNs were prepared by a procedure similar to that previously reported.^46^ Briefly, water-soluble GO and FeTCP were ultrasonically mixed to promote the π–π interaction between them, then hydrazine hydrate was added to the above solution to reduce the GO (see Methods in ESI†). As shown in Fig. 1a, a stable FGHNs dispersion was obtained (Fig. 1a, inset III) after hydrazine reduction, with the peak at 226 nm for GO, attributed to the π–π* transition of aromatic C=C bonds, shifting to 261 nm for rGO.^47^ A new absorption peak at 423 nm was also observed, which corresponds to the Soret band of FeTCP with a red shift (27 nm).^46–48^ The results indicate that FeTCP was adsorbed on the surface of the rGO sheets by π–π interactions. Meanwhile, when compared with FGHNs, the dispersion of rGO without the stabilization of FeTCP leads to the aggregation of rGO sheets after reduction (Fig. 1a, inset II). The attachment of FeTCP on the rGO surface was also characterized by an electrochemical method. Fig. 1b shows the cyclic voltammograms of bare ITO (blue line), FeTCP/ITO (purple line), rGO/ITO (red line) and FGHNs/ITO (black line) in 0.1 M phosphate buffered saline (PBS). Compared with ITO and rGO/ITO, a pair of redox peaks were observed in the potential range from –0.6 V to 0.0 V for FGHNs/ITO. The redox peaks should obviously belong only to FeTCP in FGHNs, which is characteristic of the electron transfer process of iron at the core of Fe(iii) TCP/Fe(ii) TCP (Fig. 1b, purple line). In addition, AFM results show that the average thickness of the single-layer FGHNs was determined to be about 1.45 nm (Fig. 1c). There was a 0.80 nm increment compared with that of pure rGO, the single-layer thickness of which is *ca.* 0.65 nm,^47^ owing to the presence of FeTCP on the rGO sheet surfaces. Thus the thickness of the FeTCP layer was calculated to be about 0.40 nm, since FeTCP can locate on both sides of the rGO.^49,50^

**Fig. 2:**
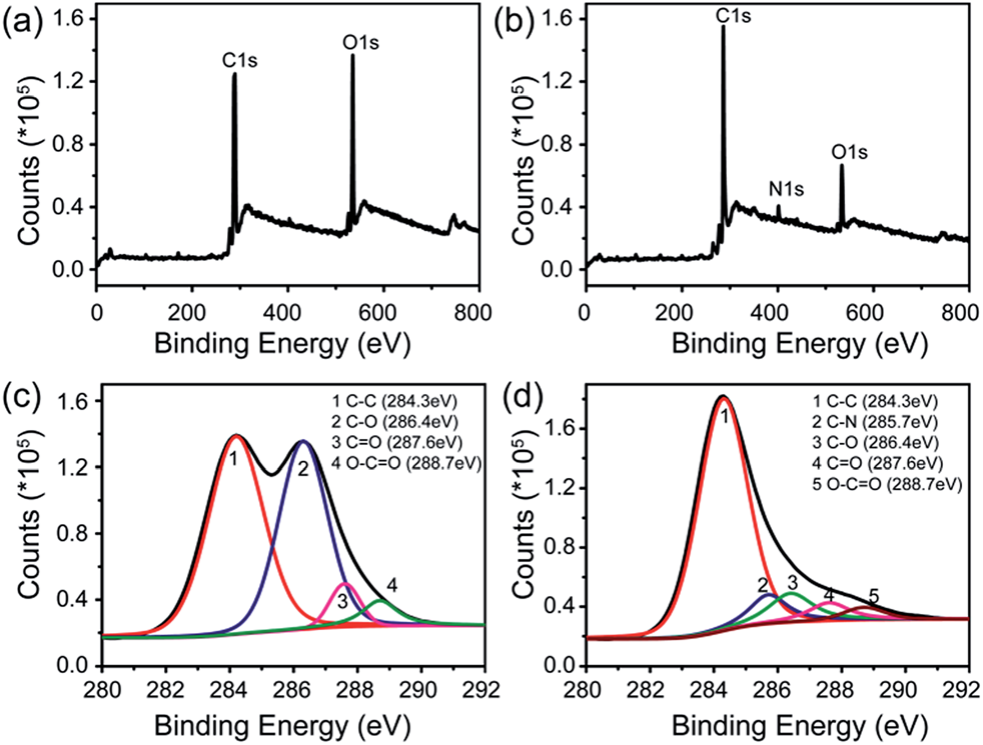
XPS data for (a) GO and (b) FGHNs. The deconvolution of C1s spectra of (c) GO and (d) FGHNs.

X-ray photoelectron spectroscopy (XPS) was employed to further explore the interaction between rGO and FeTCP. Compared with GO (Fig. 2a), the survey of FGHNs shows the presence of detectable amounts of N1s at about 400 eV, originating from FeTCP, which indicates that the functionalization of rGO by FeTCP occurred successfully (Fig. 2b). After hydrazine reduction, the C1s spectrum indicates that the peaks associated with C–C or C–H (284.3 eV) become predominant, while the peaks related to the oxidized carbon species (C–O, C=O) are greatly weakened (Fig. 2c and d). Meanwhile, a new peak appeared at 285.7 eV in the spectra of FGHNs, which belongs to the carbon in the C–N bonds. These results indicate that GO has been well deoxygenated into rGO and further protected by FeTCP molecules to form FGHNs.

### Electrochemical behaviors of FGHNs and APBA/FGHNs

**Fig. 3:**
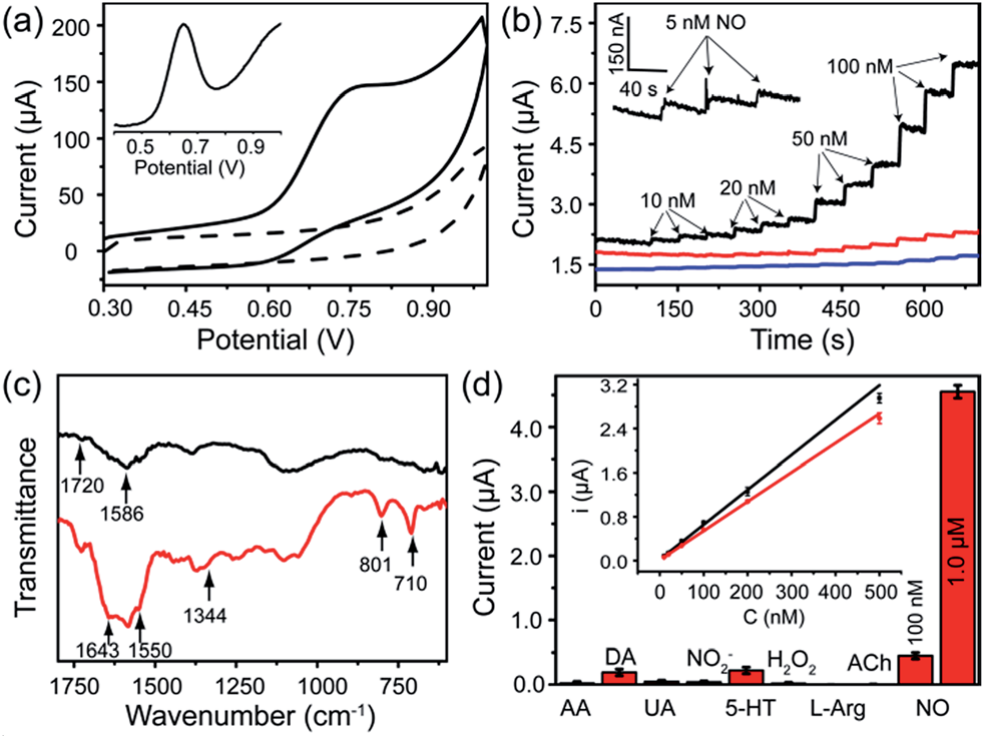
(a) Cyclic voltammograms of FGHNs/ITO in the absence (dashed line) and presence (solid line) of 10 μM NO in deaerated PBS solution at a scan rate of 50 mV s^–1^. Inset: differential pulse voltammogram of FGHNs/ITO to 10 μM NO. (b) Amperometric curves of FeTCP/ITO (blue line), rGO/ITO (red line), FGHNs/ITO (black line) to a series of NO concentration increases in a stirred deaerated PBS solution and the response of FGHNs/ITO to 5 nM NO is magnified in the inset. A potential of +0.75 V (*vs.* Ag/AgCl) was applied to all the electrodes. (c) ATR-IR spectra of FGHNs (black line) and APBA/FGHNs (red line). (d) Selective profile of the APBA/FGHNs/ITO to interferences in the cell medium RPMI 1640, in which all interfering species were at the concentration of 2 μM. Inset: calibration curves of FGHNs/ITO (black line) and APBA/FGHNs/ITO (red line) for increasing NO concentration in the cell medium.

The FGHNs/ITO exhibited excellent electrochemical behavior to NO (the standard solution was prepared as previously described^26^) and the differential pulse and cyclic voltammograms showed a peak at +0.65 V and +0.75 V (Fig. 3a), respectively. It is worth noting that FGHNs/ITO acted as the best catalyst compared to rGO/ITO and FeTCP/ITO (Fig. 3b), the sensitivities of which were calculated to be 37.6, 7.2 and 2.1 μA μM^–1^ cm^–2^, respectively. The metalloporphyrin was used as a catalytic coating for the construction of NO electrochemical sensors to improve sensitivity and specificity,^7,21,23^ but it has poor electrical conductivity. The introduction of the underlying rGO greatly enhanced the catalytic capability to NO by providing a highly conductive bridge to facilitate rapid transport of electrons between the porphyrin and the electrode.^48,50^ This attributes to the amazingly synergistic electrooxidation of NO.

FGHNs/ITO were further functionalized covalently with the small cell-adhesive molecule APBA by coupling the –COOH and –NH_2_ groups in FeTCP and APBA. Attenuated Total Reflection Infrared Spectroscopy (ATR-IR) of FGHNs displayed two peaks at 1720 and 1586 cm^–1^ (Fig. 3c, black line), which are assigned to the stretching mode of a carboxyl group in FeTCP. After the covalent bonding of APBA with FGHNs, two new peaks at 1643 and 1550 cm^–1^ corresponding to the characteristic amine groups were observed (Fig. 3c, red line). In addition, the presence of the B–O stretching mode at 1344 cm^–1^, together with the clearly aromatic C–H stretching at 801 and 710 cm^–1^, further evidenced the successful immobilization of APBA.

The comparison of electrochemical responses in PBS solution (data not shown) and the cell medium (Fig. S1†) were also investigated, and the average sensitivities of APBA/FGHNs/ITO in both conditions remained at more than 80% (the ratio of the calibration curve slopes) of FGHNs/ITO after its further functionalization and reuse (Fig. 3d, inset), indicating the additional influence of small cell-binding molecule APBA to the response of electrodes. The selectivities of FGHNs/ITO and APBA/FGHNs/ITO toward NO in PBS were studied by investigating interferents such as ascorbic acid (AA), dopamine (DA), uric acid (UA) and NO_2_^–^ (Fig. S2†). The calculated selectivity ratios for NO against AA, DA, UA and NO_2_^–^ were 89, 74, 111 and 89 for FGHNs/ITO, and 113, 96, 158 and 117 for APBA/FGHNs/ITO, indicating that FGHNs/ITO possesses good selectivity against these interferents, especially for negatively charged molecules, and APBA/FGHNs/ITO has better performance than FGHNs/ITO. The good selectivity might originate from the highly inherent specificity of the metalloporphyrin to the electrocatalytic oxidation of NO. The retained surface carboxyl groups together with boronate of APBA/FGHNs also enhanced the selectivity.

To further demonstrate the selectivity of the sensor in physiological solutions, we conducted the measurements in the cell culture medium RPMI 1640 with serum (Fig. 3d). In addition to AA, DA, UA and NO_2_^–^, other potential interferents including 5-hydroxy tryptamine (5-HT), H_2_O_2_, l-arginine (l-Arg) and acetylcholine (ACh) were tested (Fig. S3†), with 2 μM of each interferent. The sensor exhibited a practical selectivity against most molecules, except positively charged DA and 5-HT with a small detectable signal. In addition, the response of the sensor remained about 90% of the initial current after 10 measurements, indicating high reproducibility of the sensor.

### Cell adhesion and proliferation on biomimetic APBA/FGHNs film

**Fig. 4:**
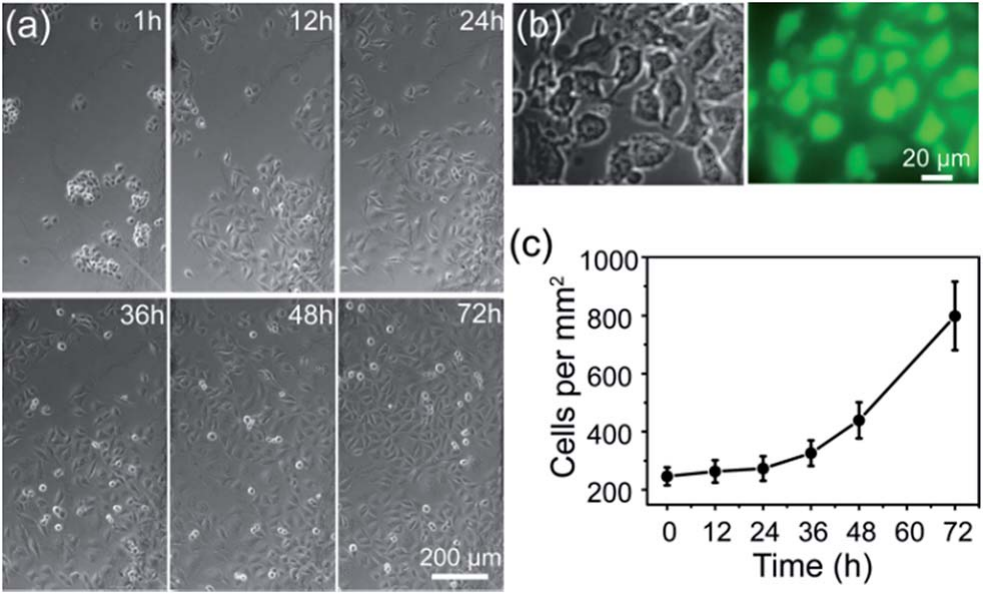
(a) Microscopic images of cells cultured on APBA/FGHNs/ITO for different times. (b) Microscopic photographs of HUVECs cultured for 86 h and labeled with Calcein-AM (green) and PI (red). (c) Proliferation curve of HUVECs cultured on APBA/FGHNs/ITO.

Construction of a cell-compatible sensing interface on which cells could be immobilized and grown directly is of great importance to detect cell-released molecules accurately, especially for those that are labile and could be metabolized rapidly, for example, free radical NO. APBA is a molecule capable of binding specifically with the 1,2- or 1,3-diols which exist in the carbohydrates of cell membranes,^42–46^ therefore it can be employed as an artificial carbohydrate-receptor for cell adhesion. After being seeded on different substrates with the same cell density and cultured for 1 h, the cells were then rinsed three times with physiological saline solution to remove loosely bound cells. It was observed that the greatest number of HUVECs were left on APBA/FGHNs/ITO (Fig. S4†), indicating the powerful cell-adhesive capacity of APBA/FGHNs. The cell proliferation behavior was also investigated by counting the number of HUVECs cultured for 1 h, 12 h, 24 h, 36 h, 48 h and 72 h (Fig. 4a), until they proliferated and covered almost all over the electrode. The cell viability was then characterized by the fluorescent live/dead cell markers Calcein-AM and PI after being cultured for up to 86 h, and cells were almost clearly alive (Fig. 4b), further displaying the excellent cytocompatibility of APBA/FGHNs.

### Microelectrode array fabrication and NO release monitoring

**Fig. 5:**
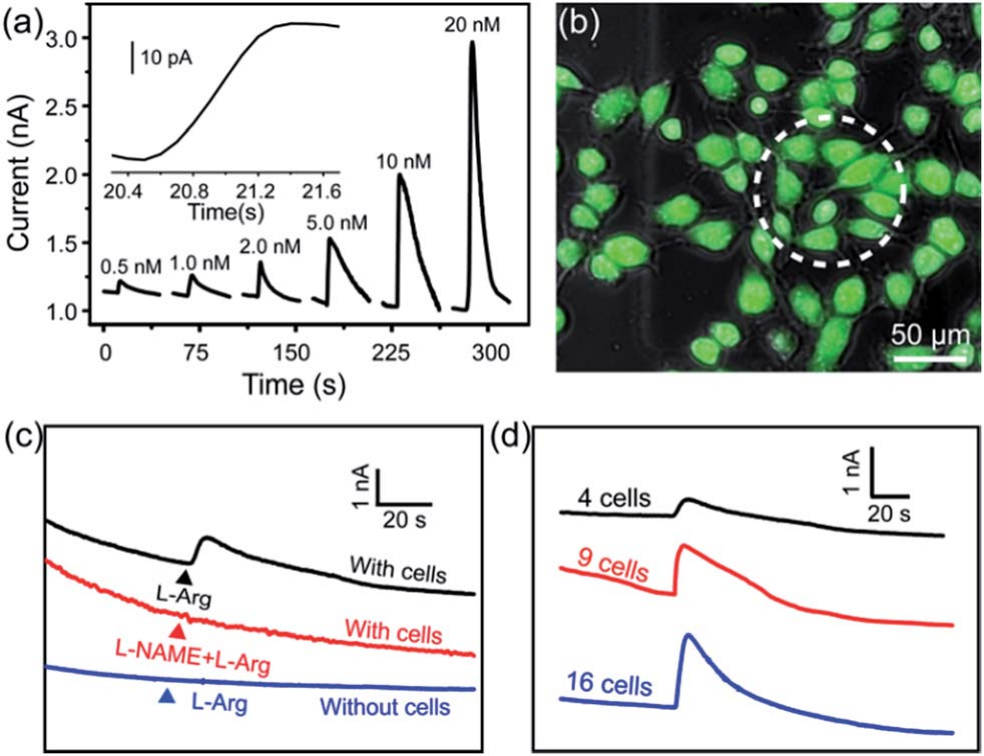
(a) Amperometric curve of APBA/FGHNs/ITO microelectrode to a series of NO concentration increases in the RPMI 1640 cell culture medium at a potential of +0.75 V. Inset: an amplified amperometric curve illustrating the response time of the sensor to NO. (b) The microscopic image of the Calcein-AM (green) and PI (red) stained HUVECs cultured on APBA/FGHNs/ITO microelectrode. Active surface area of an individual microsensor was labeled by a white dotted circle. (c) Typical response of APBA/FGHNs/ITO at different conditions. (d) Amperometric responses of microelectrodes to 4 (black line), 9 (red line) and 16 (blue line) HUVECs stimulated by l-Arg in the RPMI 1640 cell medium.

To realize the real-time monitoring of NO release at the single cell level, we fabricated a patterned ITO microelectrode (100 μm in diameter) array using photolithography techniques (see Methods section in ESI,† and an illustration of the process in Fig. S5†).^51^ The FGHNs and APBA were then deposited on the microelectrode by electrophoresis and covalent linkage, respectively (see Methods section in ESI,† and the electrode morphologies of ITO and APBA/FGHNs/ITO microsensor shown in Fig. S6†). To simulate the cell-microsensor configuration during the electrochemical detection of NO release, a fast (*ca.* 5 s) injection of different concentrations of NO solution in a micro-capillary (150 μm internal diameter, close to the active area of the electrode) was performed in PBS and the cell medium, with the results shown in Fig. S7, 5a and S8,† respectively. The sensitivity of the APBA/FGHNs/ITO microelectrode in the RPMI 1640 (0.089 nA nM^–1^, Fig. S8†) was about 70% of that in PBS solution (0.1232 nA nM^–1^, Fig. S7†), which may result from the partial adsorption of undesired compounds in the cell medium onto the active surface of the microelectrode. The typical response time of this sensor to NO was about 400 ms (Fig. S7a,† inset) and 600 ms in PBS and the cell medium (Fig. 5a, inset), if we define the response time as the interval between the instant at which current reaches 10% of the maximum and the instant at which current rises to 90% of the maximum. The fast response characteristic of the sensor facilitates the real-time monitoring of NO release from cells. The detection limit was calculated to be about 55 pM in PBS and 90 pM in the cell medium (*S*/*N* = 3), which are to the best of our knowledge the lowest of those previously reported (Table S1†).

After seeding on the APBA/FGHNs/ITO microelectrode array, the HUVECs grew and proliferated well (Fig. S9†), with a higher cell density formed on the active microelectrode surface 10 h later. Fluorescence imaging by live/dead cell markers Calcein-AM and PI demonstrated the excellent viability of the cell (Fig. 5b). Then the APBA/FGHNs/ITO microsensor was used to monitor NO release from the living cells (Fig. 5c, black line) cultured thereon. The production of NO was evoked by stimulating HUVECs with fast injection (1 μL) of 3 mM l-Arg, which can be enzymatically oxidated by nitric oxide synthase (NOS) to produce NO in endothelial cells.^52^ Control experiments were carried out to confirm the change in the measured current was due to the oxidation of NO released from HUVECs. When l-Arg was injected onto microelectrodes without cells cultured thereon or cells were simultaneously stimulated with a specific NOS inhibitor, l-NAME (100 μM), and l-Arg, no increase in current was detected (Fig. 5c, blue line and red line for the above two conditions, respectively), excluding the possibilities of l-Arg disturbance and other related electrochemical active interferences. NO release from different numbers of living cells were monitored by the sensor in both PBS (Fig. S10†) and the cell medium (Fig. 5d). Obvious increases in current were observed, which were followed by a gradual decrease of the current to the baseline, and the current amplitude was raised with the increasing number of cells cultured on the sensor. We measured the average concentration of released NO in the cell medium when all the active surface of the microelectrode was covered by HUVECs, and the quantitative value was calculated to be about 16 nM, in the range of NO released by endothelial NO synthase.^17,53^

To test the reusability of the microsensor array after cell culture and NO detection, we detached the attached cells and cell-secreted glycoprotein by firstly immersing it in 0.01 M aqueous NaOH solution followed by rinsing in water. It was observed that the attached cells could be easily detached from the sensor for subsequent cell culture and measurement owing to the pH-dependent reversible formation between boronate and carbohydrates. Electrochemical results showed that the microsensor remained at over 80% of its initial response after cell detection and sensor renewal 5 times, indicating a highly promising strategy for constructing reusable cell biosensors, especially when they are obtained by complicated microfabrication.

## Conclusions

In summary, we have constructed a multifunctional NO microsensor array based on boronic acid and metalloporphyrin co-functionalized graphene oxide. The hybrid material FGHNs exhibited excellent sensitivity to a sub-nanomolar range of NO, while APBA conferred the sensor with high cytocompatibility to cells and practicable reusability. The sensor was further used for the sensitive and selective real-time monitoring of NO molecule release from attached human endothelial cells in a cell culture medium. In addition, the sensor was transparent and could be coupled to optical imaging techniques. Though detection of NO at very low bioactive concentrations under *in vivo* physiological conditions has not yet been demonstrated , the exceptional sensitivity, excellent cytocompatibility and reusability of this sensor make it a promising sensing interface to be incorporated into integrated microfluidic or implantable devices. This can therefore facilitate the real-time monitoring of NO at extremely low concentrations in diverse physiological and pathological conditions with high spatiotemporal resolution.
