# Supplementary material for: Functionalized graphene-based biomimetic microsensor interfacing with living cells to sensitively monitor nitric oxide release
Source: Chem Sci. 2015 Jan 28;6(3):1853–8. doi: 10.1039/c4sc03123g (PMC5486208; doi:10.1039/c4sc03123g)
Supplement: Supplementary file 1 [file SC-006-C4SC03123G-s001.pdf]

Electronic Supplementary Material (ESI) for Chemical Science.
This journal is © The Royal Society of Chemistry 2015

Supporting Information for Functionalized graphene-based biomimetic microsensor interfacing with living cells to sensitively monitor nitric oxide release

Yan-Ling Liu, Xue-Ying Wang, Jia-Quan Xu, Chong Xiao, Yan-Hong Liu,

*Xin-Wei Zhang, Jun-Tao Liu and Wei-Hua Huang**

Key Laboratory of Analytical Chemistry for Biology and Medicine (Ministry of Education), College of Chemistry and Molecular Sciences, Wuhan University, Wuhan, 430072, China

E-mail: whhuang@whu.edu.cn

RECEIVED DATE:

Phone: (86)2768752149

Fax: (86)2768754067

METHODS

Materials. ITO conductive glass (film thickness: 180 nm, conductivity: 10 Ω/sq) was purchased Crystal Great Technology Co., Ltd (Shenzhen, China). Graphene oxide (GO) was obtained from Ji Cang Nano Technology Co., Ltd. (Nanjing, China). Fe (III) meso-tetra (4-carboxyphenyl) porphyrin (FeTCP) was purchased from Frontier Scientific, Inc. Hydrazine solution (85%) and ammonia solution (25 wt %) were provided by Beijing Chemicals Inc (Beijing, China). The cell culture medium RPMI 1640, L-glutamine and HEPES for HUVECs culture were purchased from GIBCO (USA). NOS inhibitor Nω-nitro-L-arginine methyl ester hydrochloride (L-NAME), N-(3-dimethylaminopropyl)-N'-ethylcarbodiimide (EDC), N-hydrosulfosuccinimide sodium salt (NHS), 3-aminophenylboronic acid (APBA) and L- arginine (L-Arg) were purchased from Sigma (USA). The human umbilical vein endothelial cell (HUVEC) lines were obtained from CHI Scientific, Inc (Shanghai China). 3',6'-Di(o-acetyl)-4',5'-bis[N,N-bis(carboxymethyl)-aminomethyl] fluorescein, tetraacetoxymethylester (Calcein-AM) and 3,8-diamino-5-[3-(diethylmethylammonio) propyl]-6-phenylphenanthridinium diiodide (PI) for cell staining were obtained from Dojindo laboratory (USA). All other chemicals unless specified were reagent grade and used without further purification.

Preparation of FGHNs and rGO. FGHNs was prepared similarly to the literature.[1] Briefly, 4 mg GO and 6.5 mg FeTCP powder were ultrasonically dissolved in 8 ml H_2O for 1 h. The whole solution was then vigorously stirred at 70 ℃ overnight.

Subsequently, 4 μL hydrazine solution and 50 μL ammonia solution were added to the above solution and the mixture was put in a water bath (95 ℃) for 1 h under agitation. The resulting product was stable black dispersion, subsequently filtered through a Nylon membrane (0.22 μm) and thoroughly washed with water until the pH of the filtrate reached 7.0. The as-prepared nanocomposite was ultrasonically dispersed in ultrapure water to obtain a FGHNs solution (0.1 mg/mL). Additionally, the preparation of rGO solution was similar to FGHNs except no addition of FeTCP.

Fabrication of ITO microelectrode array. The ITO film (5 cm × 5 cm) was patterned into 200-μm wide stripes terminating in 1 by 10 mm rectangles to allow connection to conducting lines. Photolithography and wet-etching techniques were adapted to pattern ITO (Figure S3) according to previous literature.[2] Briefly, AZ 4620 photoresist (Clariant, USA) was spin-coated on the ITO-glass slide at 3000 rpm and baked at 65 ℃ for 3 min and then 105 ℃ for 5 min on a hot plate. It was next exposed under UV light using a high-resolution (20000 dpi) transparency film as a photomask. The photoresist was then developed in the recommended developer solution. Concentrated hydrochloric acid was used to etch the portion of the ITO that was not protected by photoresist. The photoresist covered on ITO electrodes was exposed through the designed insulating mask using microscope for mask alignment and the photoresist was developed, as above. After thoroughly rinsed with ultrapure water and dried with N_2 gas, insulating layer Si_3N_4 was deposited by magnetron sputtering techniques. The undeveloped photoresist was then removed by “lift-off” with acetone, revealing the active area and wiring part. Besides, a PDMS frame (1 cm × 1 cm),

designed as cell culture chamber, was made by casting PDMS prepolymer on the ITO electrodes and curing at 75 ℃ for 1 h.

Modifying ITO microelectrode array with APBA/FGHNs. FGHNs was immobilize onto ITO microelectrodes array by electrophoretic deposition. Briefly, 200 μL FGHNs solution (0.1 mg/mL) was drop into the PDMS chamber. The ITO microelectrode (100 μm in diameter) and a platinum wire were employed as the positive electrode and the negative electrode, respectively. Under a constant potential of +3 V for 300 s, the FGHNs films were deposited onto ITO microelectrodes from the aqueous solution of FGHNs. After the rest solution was removed, the ITO was repeatedly washed with deionized water and dried at room temperature. The ITO microelectrodes were immersed in a solution of 4 mM EDC for 0.5 h, then 10 mM of NHS was added for activation. 1 h later, 10 mg APBA was then added and the solution was slightly stirred to dissolve APBA. After left overnight at room temperature, ITO microelectrodes were rinsed with pH 7.4 phosphate buffer.

Human umbilical vein endothelial cell culture and manipulation. Human umbilical vein endothelial cells (HUVECs) were routinely cultured using RPMI 1640 culture medium with 12% fetal bovine serum, 0.292 mg/mL L-glutamine, 4.766 mg/mL HEPES, 0.85 mg/mL $NaHCO_3$, penicillin and streptomycin (100 U) in the culture flask at 37 ℃ in a humidified incubator (95% air with 5% CO_2). For cells detection, HUVECs were seeded on APBA/FGHNs/ITO microelectrode at a density of ~1×10^6 cell/cm^2. It was kept in the incubator for 1 h to allow cells to be adhered

onto the microelectrode and loosely bounded HUVECs were washed away. Cells cultured for 10 h were chosen for NO release study.

Apparatus and characterization. AFM image was taken by a SPM-9500J3 microscope (Shimadzu, Japan) operating in the tapping mode with standard silicon nitride tips, the sample was obtained by drop-casting diluted FGHNs solution onto freshly cleaved mica. XPS measurement was performed on an XSAM800 photoelectron spectrometer (Kratos, UK) with Al Kα X-ray radiation as the X-ray source for excitation. UV-vis absorption spectra were recorded on a UV-3600 spectrophotometer (Shimadzu, Japan). ATR-IR spectra were measured on a FIRT iS10 spectrometer (Thermo, USA). Electrochemical measurements were carried out on CHI660A electrochemical workstation (CHI Instruments, Shanghai). A three-electrode system was used in the experiment including a bare or modified ITO working electrode (0.5 cm × 0.5 cm), Ag/AgCl reference electrode and Pt counter electrode. A two-electrode system was adopted in microelectrode arrays test with ITO microelectrodes (100 μm in diameter) and an Ag/AgCl electrode. An Axiovert 200M inverted fluorescent microscope (Zeiss, Germany) was utilized for observation and a TransferMan NK2 micromanipulator (Eppendorf, Germany) was employed for NO and stimulus solution injection.

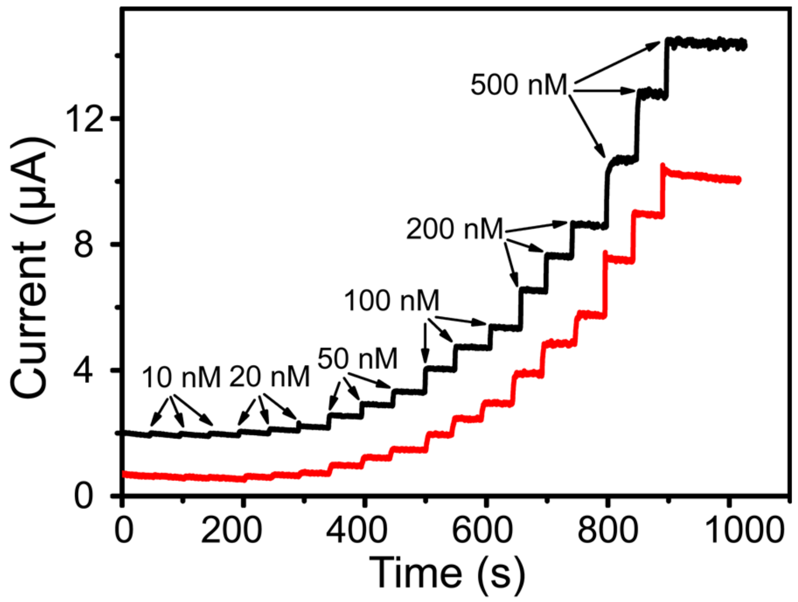

Fig S1 Amperometric response curves of the FGHNs/ITO electrode (black line) and after its functionalization with APBA (red line) for a series of NO concentration increases in a stirred cell medium.

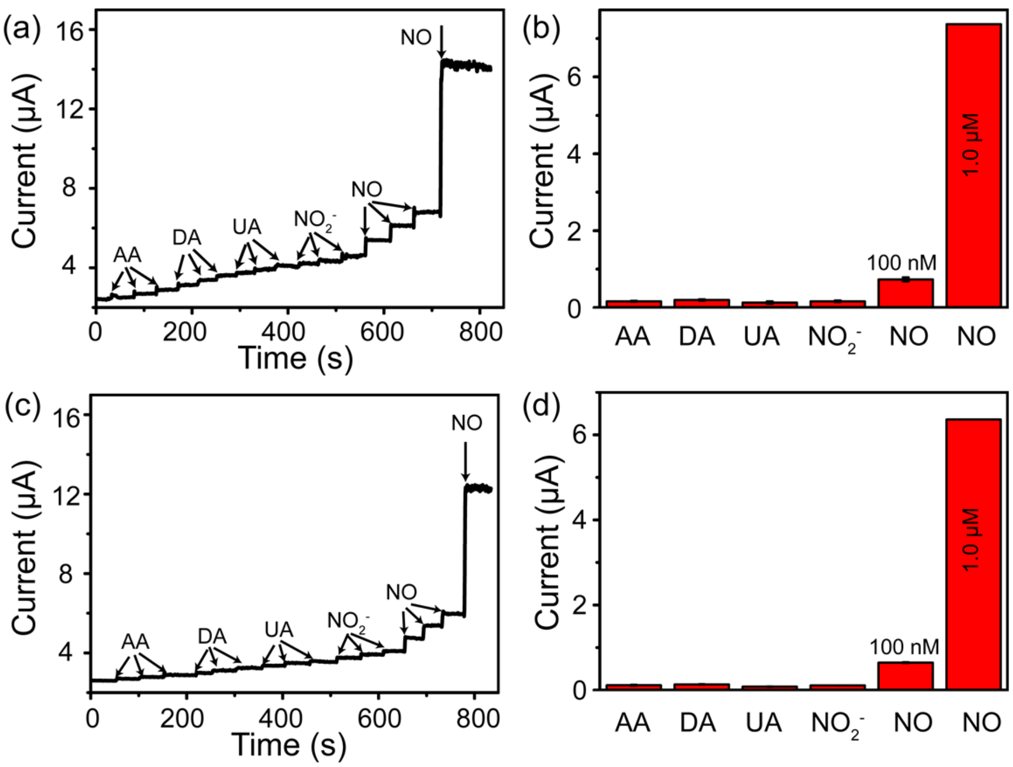

Fig S2 Amperometric response curves of FGHNs/ITO (a) and APBA/FGHNs/ITO (c) electrodes to a series of interferences (2 μM) and NO solutions (100 nM and 1 μM) in a stirred deaerated PBS solution, and the corresponding selective profiles of FGHNs/ITO (b) and APBA/FGHNs/ITO (d).

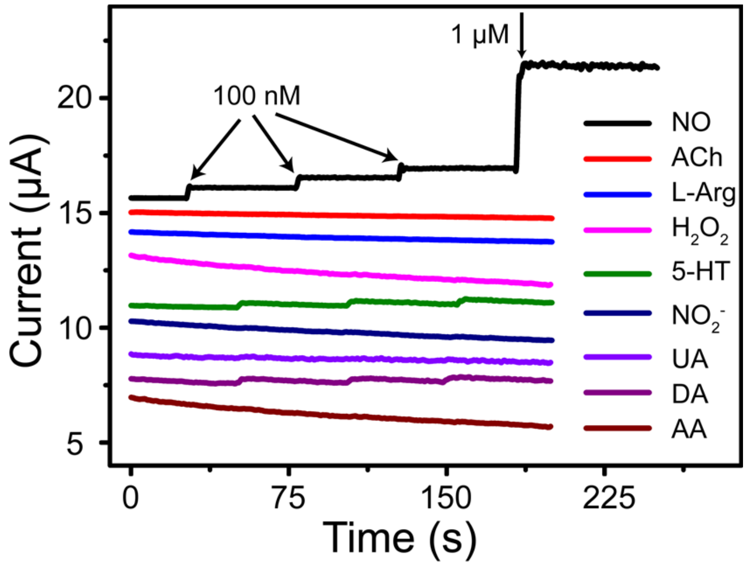

Fig S3 Amperometric response curves of APBA/FGHNs/ITO electrodes to a series of control (AA, DA, UA, NO_2- 5-HT, H_2O_2, L-Arg and Ach, the concentration of each interferent was 2 μM) and NO solutions in RPMI 1640 cell culture medium at a potential of +0.75 V.

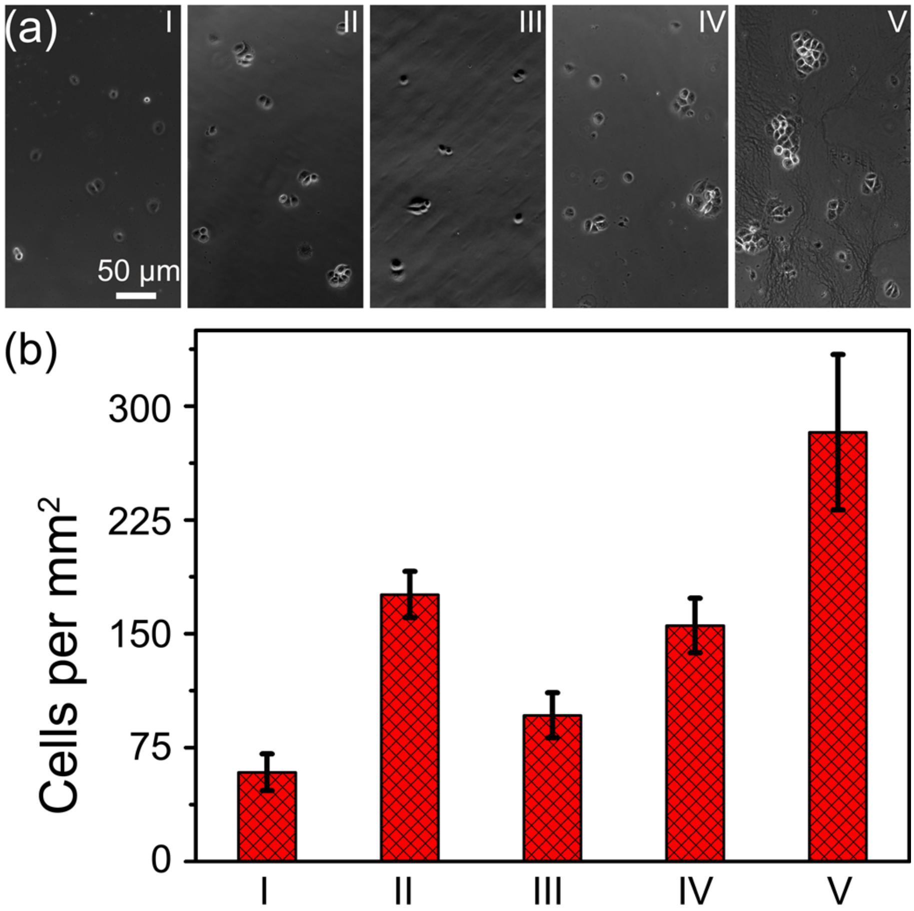

Fig S4 (a) Microscopic images of HUVECs left on ITO (I), rGO/ITO (II), FeTCP/ITO (III), FGHNs/ITO (IV) and APBA/FGHNs/ITO (V) after cultured for 1 h and then washed with PBS solution for 3 times and (b) the corresponding statistical data of cell numbers on different substrates (n=8).

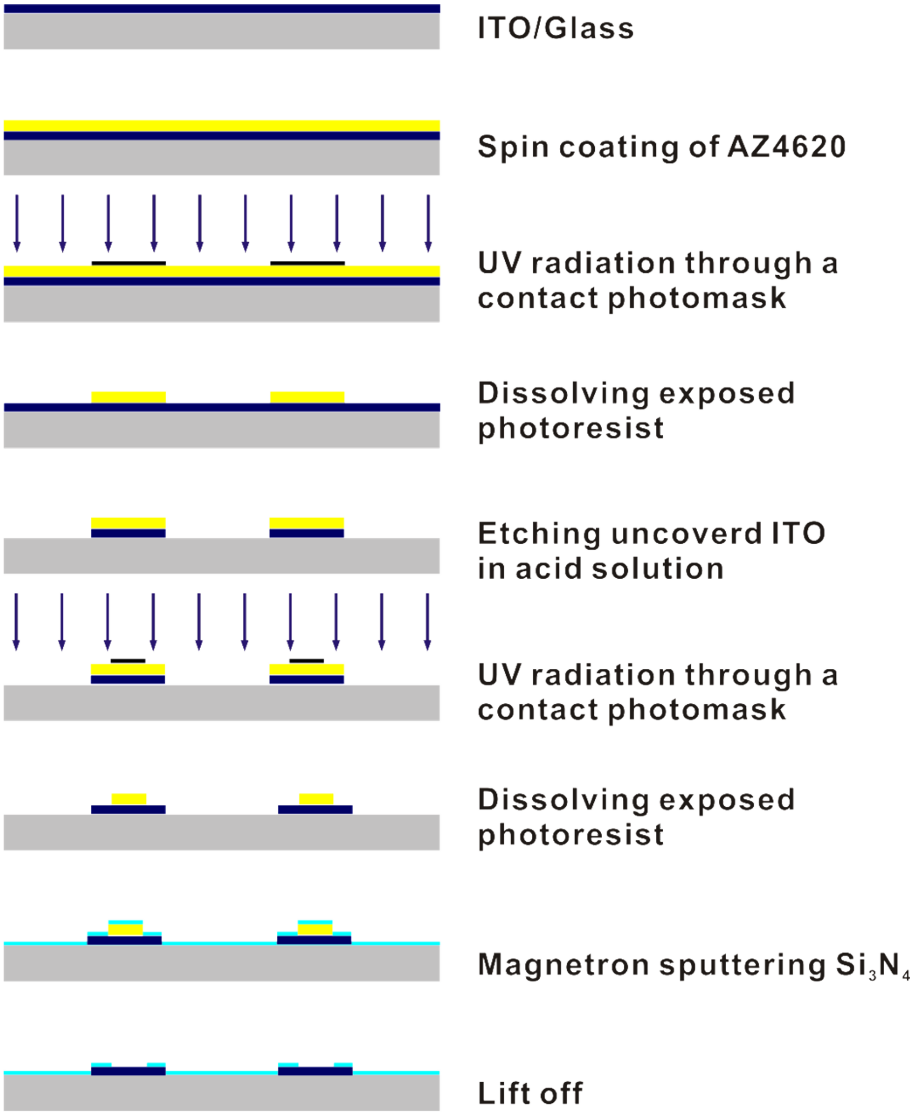

Fig S5 Schematic diagram demonstrating the fabrication procedures of ITO microelectrode array.

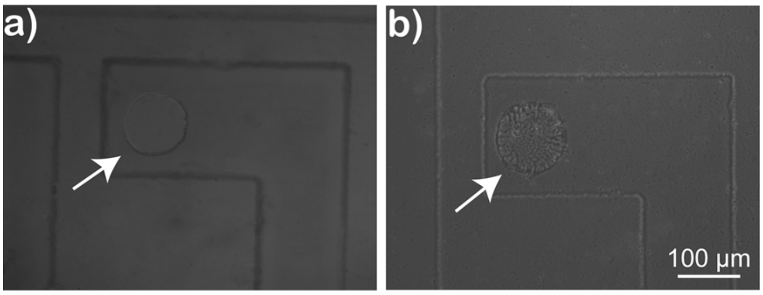

Fig S6 Microscopic images of an individual ITO microelectrode before (a) and after modification with APBA/FGHNs (b). The circular area in each image the arrow pointed to was the active electrode surface.

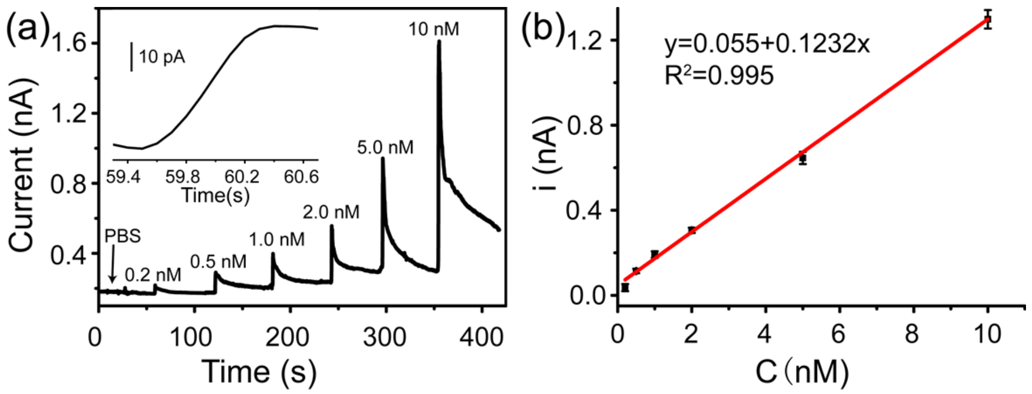

Fig S7 (a) Amperometric response curve and (b) the calibration curve of the sensor to a series of NO concentration increases in PBS solution.

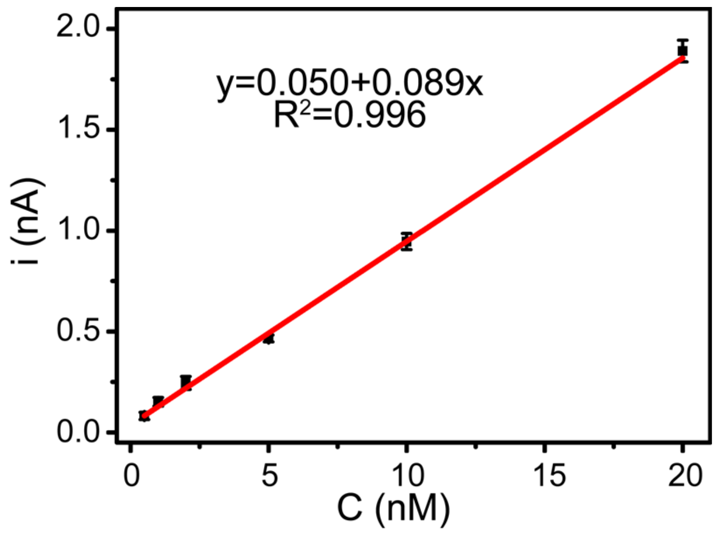

Fig S8 The calibration curve of the sensor to a series of NO concentration increases in cell medium.

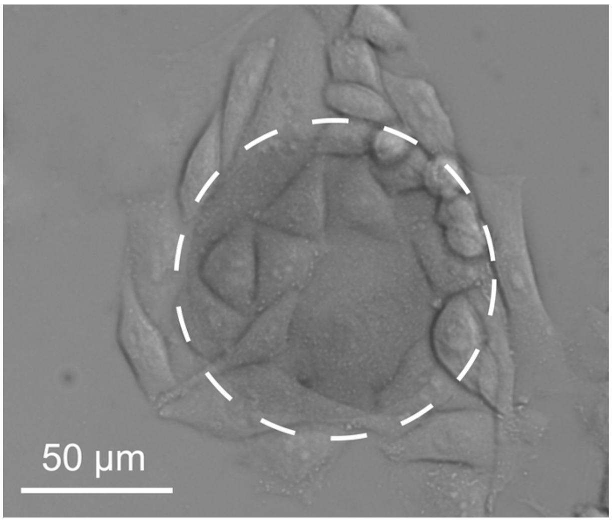

Fig S9 Microscopic image of HUVECs cultured on patterned APBA/FGHNs/ITO microelectrode for 10 h.

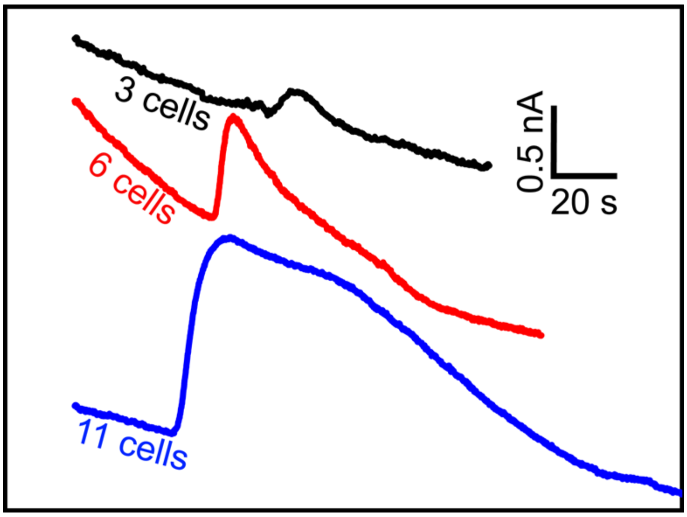

Fig S10 Amperometric responses of microelectrodes at a potential of +0.75 V to 3 (black line), 6 (red line) and 11 (blue line) HUVECs stimulated by L-Arg.

Table1. Materials and performance characteristics of selected NO electrochemical microsensors in PBS solution.

Electrode	Modifying materials	Diameter (μm)	LOD (nM)	Reference
Carbon fiber	poly-TMHPP-Ni/Nafion	0.5	10	3
Pt	polystyrene	10	75	4
Carbon fiber	WPI membrane/Nafion	0.1	2	5
Pt/Pt black	PTFE	250	1	6
Pt/Pt black	Fluorinated xerogel	20	0.083	7
pAu/ITO	Au- Prussian Blue/PTFE	NA	~1	8
ITO	APBA/FGHNs	100	0.055	This work

LOD, limit of detection. TMHPP, tetrakis (3-methoxy-4-hydroxyphenyl) porphyrin. PTFE, polytetrafluoroethylene. pAu, porous Au. NA, not available

References

1. L. Feng, L. Wu, J. Wang, J. Ren, D. Miyoshi, N. Sugimoto, X. Qu, *Adv. Mater.*, 2012, **24**, 125-131.
2. W. Zhan, J. Alvarez, R. M. Crooks, *J. Am. Chem. Soc.*, 2002, **124**, 13265-13270.
3. T. Malinski, Z. Taha, *Nature*, 1992, **358**, 676-678.
4. Y. Kitamura, T. Uzawa, K. Oka, Y. Komai, H. Ogawa, N. Takizawa, H. Kobayashi, K. Tanishita, *Anal. Chem.*, 2000, **72**, 2957-2962.
5. X. Zhang, Y. Kislyak, J. Lin, A. Dickson, L. Cardosa, M. Broderick, H. Fein, *Electrochem. Commun.*, 2002, **4**, 11-16.
6. Y. Lee, J. Kim, *Anal. Chem.*, 2007, **79**, 7669–7675.
7. J. H. Shin, B. J. Privett, J. M. Kita, R. M. Wightman, M. H. Schoenfisch, *Anal. Chem.*, 2008, **80**, 6850–6859.
8. W. Cha, Y.-C. Tung, M. E. Meyerhoff, S. Takayama, *Anal. Chem.*, 2010, **82**, 3300-3305.
